# Supplementary material for: Annexin A1 Is Associated with Adverse Clinical Outcomes in Patients with COVID-19
Source: J Clin Med. 2022 Dec 17;11(24):7486. doi: 10.3390/jcm11247486 (PMC9781714; doi:10.3390/jcm11247486)
Supplement: Supplementary file 1 [file jcm-11-07486-s001.zip › jcm-2042393-supplementary.pdf]

SUPPLEMENTALS

Table S1. Median AnxA1 (interquartile range) of moderate and severe patients with COVID-19 during the course of disease. AnxA1 did not significantly change over time between moderate and severe patients.

	Moderate		Severe		p-value
AnxA1, ng/mL	*n*	*median (IQR)*	*n*	*median (IQR)*	
Baseline	68	30.1 (16.0-42.0)	104	28.9 (17.3-53.6)	0.583
≤ 7 days	31	27.3 (13.8-49.9)	65	39.1 (23.4-60.3)	0.102
8 – 14 days	9	37.5 (32.0-55.2)	50	39.6 (27.4-64.8)	0.891

Differences between groups were analyzed by the Mann Whitney U test.

Abbreviation: AnxA1, Annexin A1.

Table S2. Baseline characteristics of patients with normal and elevated AnxA1.

	**Normal Range**	**Normal AnxA1** (*n*=135)	**Elevated AnxA1** (*n*=85)	**Overall *P***
M/F		90/45	54/31	0.634
Age, yr.		67 (±14)	70 (±12)	0.098
Days from illness onset		7 (5-10)	9 (7-14)	**<0.001**
Vitals				
SBP, mmHg		132 (±21)	142 (±23)	**<0.001**
DBP, mmHg		79 (±13)	81 (±13)	0.305
Heart rate, bpm		90 (80-103)	86 (77-100)	0.095
Body temperature, °C	≤37.9	38.0 (±1.0)	38.0 (±1.0)	0.818
Fever, n (%)		69 (55)	37 (49)	0.002
Medical history				
Hypertension, n (%)		48 (36)	28 (33)	0.691
Diabetes, n (%)		26 (19)	21 (25)	0.337
CVA, n (%)		17 (13)	13 (15)	0.570
Cardiac disease, n (%)		44 (33)	24 (28)	0.496
COPD/asthma, n (%)		20 (15)	13 (15)	0.923
None, n (%)		31 (23)	23 (27)	0.492
Disease severity				
Mild, n (%)		37 (27)	11 (13)	**0.011**
Moderate, n (%)		40 (30)	28 (33)	0.605
Severe, n (%)		58 (43)	46 (54)	0.107

Continuous variables were presented as mean (±standard deviation) or median (interquartile range) as appropriate. Differences between groups were analyzed by the unpaired sample *t* test or Mann Whitney U test. Differences in categorical variables were analyzed by the chi square test.

Abbreviation: AnxA1, Annexin A1. SBP, systolic blood pressure. DBP, diastolic blood pressure. CVA, cerebrovascular accident. COPD, chronic obstructive pulmonary disease.

Table S3. Correlations between AnxA1 and inflammatory markers and vWF:Ag at baseline in patients with COVID-19.

	**AnxA1 (ng/mL)**	
	Spearman's *r*	*P* value
**CRP (mg/L)**	0.227	**0.001**
**Leukocytes (×109/L)**	0.309	**<0.001**
**Neutrophils ($x10^9$/L)**	0.297	**<0.001**
**Lymphocytes ($x10^9$/L)**	0.160	**0.026**
**NLR**	0.082	0.457
**C5a (ng/mL)**	0.190	**0.008**
**vWF:Ag (%)**	0.315	**<0.001**

Spearman rank correlation coefficients were used.

Abbreviation: AnxA1, Annexin A1. CRP, C-reactive protein. NLR, neutrophil-lymphocyte ratio. C5a, complement 5a. vWF:Ag, von Willebrand factor antigen.

Table S4. Univariable regression models for predictors of thrombotic events, ICU admission, and 28 days mortality in patients with COVID-19.

Thrombotic events. Male sex and the risk for developing a thrombotic event during the course of COVID-19 (OR 4.867 [95% CI 1.415-16.733]; p < 0.012).

ICU Admission. Sex (male) (OR 2.163 [95% CI 1.117-4.189]; p = 0.022), CRP (mg/L) (OR 1.008 [95% CI 1.005-1.012]; p < 0.0001) and hypertension (OR 0.462 [95% CI 0.239-0.895]; p = 0.022) and cardiac disease (OR 0.455 [95% CI 0.228-0.907]; p = 0.025) as comorbidities were risk factors for ICU admission in patients with COVID-19.

28 days mortality. Sex (male) (OR 2.056 [95% CI 1.023-4.131]; p = 0.043), CRP (mg/L) (OR 1.004 [95% CI 1.001-1.008]; p = 0.015) and diabetes as comorbidity (OR 3.551 [95% CI 1.771-7.120]; p < 0.0001) were risk factors for 28 days in-hospital mortality in patients with COVID-19.

Table S5. Linear mixed models calculated mean predicted AnxA1 levels at baseline and over time for thrombotic events, ICU admission and 28 days in-hospital survival.

	**AnxA1**	
**Thrombotic events**	*Estimate (95% CI)*	p-value
Thrombosis	+ 27.8 (-2.7-58.3)	0.074
Over time	+ 5.4 (0.1-10.7)	**0.048**
Thrombosis*Over time	- 3.2 (-13.6-7.2)	0.537
**ICU admission**	*Estimate (95% CI)*	p-value
ICU admitted	+ 20.5 (-2.6-43.5)	0.082
Over time	+ 2.8 (-5.0-10.5)	0.480
ICU admitted*Over time	+ 2.1 (-7.6-11.7)	0.670
**28 Days Mortality**	*Estimate (95% CI)*	p-value
Non-survivors	+ 7.7 (-17.9-33.3)	0.553
Over time	+ 5.0 (-0.1-10.2)	0.056
Non-survivors*Over time	- 1.4 (-12.4-9.7)	0.806

Abbreviation: AnxA1, Annexin A1. ICU, intensive care unit.
